# To Create a Safe and Healthy Place for Children: The Associations of Green Open Space Characteristics With Children's Use

**DOI:** 10.3389/fpubh.2021.813976

**Published:** 2022-03-16

**Authors:** Ming Ma, Michael Adeney, Wei Chen, Darong Deng, Shaohua Tan

**Affiliations:** ^1^College of Architecture and Urban Planning, Yunnan University, Yunnan, China; ^2^Institute for Smart City of Chongqing University in Liyang, Chongqing University, Chongqing, China; ^3^Faculty of Architecture and Urban Planning, Chongqing University, Chongqing, China; ^4^School of Public Health, University of Washington, Seattle, WA, United States; ^5^Department of Bone and Joint Surgery, Sanya Central Hospital, Sanya, China; ^6^The First Studio, Chongqing Planning and Design Institute, Chongqing, China

**Keywords:** green open space, children, physical activity, design, planning, landscape, environmental characteristics

## Abstract

Green open space (GOS) is an important outdoor resource for the well-being of children by providing places for physical activity (PA), especially in the highly urbanized environment. The COVID-19 lockdowns have made children have more sedentary time than before due to less access to public places. This article aims to examine the associations of GOS characteristics (environmental and surrounding) and children's use (visitation and PA pattern) to provide evidence for promoting their PA during the pandemic. This study employed the method of GPS positioner, accelerometer, and survey to measure the children's actual use in GOS. A total of 179 children participated in the study and 10 GOSs were selected. The children were provided with the accelerometers and GPS positioners to track their walking steps, duration, and locations. The environmental characteristics and 1 km buffer of the selected GOSs were explored as extended study area. Results showed that 49.16% of children reported more visitations than before the pandemic, and 48.60% of them preferred to go on weekdays during the pandemic. Both environmental and surrounding characteristics could affect the visitation pattern. The size (*p* < 0.000), residential ratio (*p* < 0.000), and intersection density (*p* < 0.000) were found as the factors significantly associated with visitation pattern. The children's PA pattern was mainly associated with the environmental characteristics of size (*p* < 0.000), sports, and playground proportion (*p* < 0.000). The locations of children's PA were mainly around square, playground, sheltered place, and waterside areas. COVID-19 has transformed the children's use of GOS, as well as their relationship with GOS. The large GOS was more likely to promote PA and its use by the children. The environmental and surrounding characteristics of GOS could affect their use pattern, whereas their PA pattern was mainly associated with the environmental characteristics. The findings suggest that GOS characteristics could be an effective solution to respond the challenge from the pandemic, and promote their visitation and PA.

## Introduction

COVID-19 has substantially changed the relationship of the human life and urban environment since its first outbreak in Wuhan, China, in December 2019. People had to follow the practice of social distancing and “self-quarantine,” and one half of the global population was required to stay at home, resulting in a negative influence to their health (1). One of these consequences is the decrease in physical activity (PA) among children, which is a major cause of overweight and obesity (2). PA is a health-prompting behavior, which is important for children's physical and mental growth, especially when it comes to moderate to vigorous physical activity (MVPA) (3). Before COVID-19, reports showed that less children meet the recommended level of PA (4, 5). The pandemic aggravated this trend due to the closures of school, gyms, parks, and other public places. The outdoor play and walking decreased significantly after pandemic restrictions were posed (6, 7). Children living in the higher dwelling density and closer to the roads were more likely to be impacted (8). Children were reported to have low PA level, less outdoor time, and higher sedentary lifestyle during the lockdown (9). According to a report from Canada, <3% of children could reach the PA guidelines due to the closure of common indoor and outdoor places (10). Some studies suggested the importance of green open space (GOS) in the pandemic, which could alleviate residents' negative effect of lockdowns by providing places for exercising and relaxing while ensuring the safety (1, 11, 12). Some GOSs were reported with increasing visitation because staying outdoors was thought be safer and healthier than indoors during the pandemic (13, 14). Some studies showed that GOS could be essential to the low-income population or those living in the highly dense urban environment because they are not likely to have private green space to mitigate the influence from the pandemic (13). It is necessary to reconsider the relationship of GOS and people, whereas few of the studies have explored the actual effect of GOS on the PA of children.

Green open space is a public place and an open space with abundance of natural and green features including park, greenway, green infrastructure, etc. Except for the green attribute, it is also featured with environmental components and recreational functions that can contribute to human health for various dimensions (15–17). For children population, studies have revealed the positive relationship between GOS and PA (18, 19). GOS was assumed as the primary outdoors setting for children on encouraging PA and producing positive health outcomes (20, 21). Understanding how children use and play in different GOS could support effective interventions to promote PA for health benefit on various layout, size, and location (22). In highly urbanized areas, GOS is the one of the most important environmental solutions for improving PA because they offer facilities and programming specifically for children from low socioeconomic status (23, 24). Longitudinal studies proved the health potential of GOS for children by promoting physical activities and reducing their BMI (25, 26). Moreover, the green and natural settings could alleviate children's negative moods and restore their direct attention when using GOS (27). The social engagement would also be promoted by the active exposure to GOS (28). Overall, there is abundance of evidence showing the importance of GOS to promote the PA and well-being of children. Nonetheless, how to respond to the challenges from COVID-19 by understanding the effect of GOS on children's use is still unclear.

The availability and characteristics of GOS are associated with children's use, meanwhile its built environment features are related to children's PA (29). The GOS characteristics consists of the physical design and planning of the urban environment, such as land use pattern and transportation system. The environmental characteristics of GOS were found to be influential on children's use such as the landscape features, shade cover, and environmental quality (30–32). Their use of GOS was associated with the presence and a variety of active recreation facilities, size of fields, and level of maintenance (33). Their perceptions of the GOS availability and environmental quality were reported to affect their use and PA level (34). Some studies examined the GOS-based PA regarding the surrounding neighborhood environment (35), showing that neighborhood with high walkability, high level of land use mixture, transit density, and destinations of parks and recreation facilities, had a great potential to promote PA among children. In another side, parents' perception of safety and access to mixed land use (36, 37), residential density, recreational facilities, and open space could make a great impact on the children's PA (31, 38). The street density could affect children's PA by perceiving the traffic as a safety barrier to hinder the GOS visitation of children (39, 40). The accessibility, sidewalk condition, connectivity of street around GOS were all reported to associate with children's PA (41, 42). However, few studies examined the effect of GOS on children's PA using multilevel factors from the environmental and neighborhood characteristics.

In spite of the recognition that the GOS characteristics are important for the children's well-being, by affecting their PA, the knowledge is still limited about assessing the children's actual use of GOS and its relationship with GOS during the pandemic. Specifically, there is also a lack of studies focusing on the specific information of children's use (such as locations, steps, and duration), which makes the evidence of promoting PA *via* GOS less practical. Moreover, studies on GOS characteristics usually just examined a few of factors on a certain aspect, which is difficult to draw a comprehensive conclusion. Given the accumulating evidence and imperious demand from the pandemic, it is critical to understand the relationship of GOS characteristics and children's use. This work aims to investigate the associations of GOS characteristics (environmental and surrounding), and children's use (visitation and PA pattern) to provide evidence for promoting their PA during the pandemic. Specific aims were: (1) Examine children's visitation and PA pattern of GOS during COVID-19; (2) Examine the extent to which GOS characteristics are associated with children's PA; (3) Identify the key factors of GOS characteristics affecting children's use. Eventually the findings would provide evidence and insights for the decision making and planning process of GOS.

## Methods

### Study Setting

Data in this work comes from a crosssectional study of children's use in 10 GOSs and their surrounding neighborhoods, in Suzhou city, China. All the GOSs are located in the highly dense urban environment, where the GOS is crucial for outdoor activity of children during COVID-19, because most of resident over there don not have private green space. The GOSs include parks, affiliated green space, and greenway, which allows residents to use without any cost. To better understand the specific effect, the children's use would be divided into two parts: (1) use visitation pattern (frequency, time, comparison), measured by site-based survey; (2) PA pattern (waking steps, duration and locations), measured by GPS positioner and accelerometer. To comprehensively understand the built environmental features, GOS characteristics consist of environmental and surrounding characteristics. The research design is trying to provide a holistic understanding of the relationship between GOS characteristics and children's use.

### Measurement

#### Children's Use

Children's use consists of visitation and PA pattern. The visitation pattern refers to how children perceive to visit and use GOS. It includes:(1) frequency they visit the studied GOS; (2) comparison with the visitation prior to COVID-19; (3) the day they prefer to visit (weekday or weekend). The visitation pattern is collected by site-based survey.

Regarding the PA pattern in GOS, a combination method of GPS positioner and accelerometer is employed. Accelerometer is used to measure the walking steps and duration, whereas GPS positioner is used to record the locations of the participants. This approach is proved the validity to track geo-data of children's PA in a given setting (43). Intensity of PA is usually measured by computing the metabolic equivalents (44), but it was not considered in this work because it was difficult to objectively calculate the intensity with high accuracy for a short period. The accelerometer (ActiGraph GT3X+) could track the walking steps and duration with high accuracy, and is widely applied in the studies of sports and physical activities for children and adolescents (45). The GPS positioner (Newman K2A) is adopted for its high accuracy (error was <5 m, outdoors) and could be initiated in a minute. It could collect the geo-data of users every 10 min and last for 8 h per full charge, which could cover the physical activities of all the children. Both of the devices could be belted in the waist of participants, without hampering their activities.

#### GOS Characteristics

The GOS characteristics consist of environmental and surrounding characteristics, which are used to describe the built environmental features inside and outside GOS. Based on the previous study and polit survey, the environmental characteristics could be summarized as size, greenery (grassland, woods, and other natural features), facilities, and amenities (square, seating, shelter, restroom, picnic area, etc.), sports and playground (sports courts and field, swing sets, splashpads, playground equipment, and other recreational places). To mediate the impact of size of GOS, all these variables are measured by the proportion instead of actual areas. These factors could be objectively measured by calculating the area of the polygon enclosing the areas, then merged and processed with geo-data of children's PA. Regarding the surrounding characteristics, four parameters are selected as residential ratio, intersection density, transit density, and land use mixture, which are proved to be constantly related to the PA of children (46). The scope of surroundings was defined as the area within 1 Km along the street network and includes all the parcels with access to the GOS boundary within 1 km. This distance was reported to be the threshold for children's walking range as well as parents would allow due to the safety concern (47, 48). The residential ratio, land use mixture, and transit density are calculated and drawn based on the official documents of zoning and planning from the Bureau of Natural Resources and Planning in Suzhou. The intersection density is measured by the number of street and road intersections, which indicate the connectivity around the studied GOS.

#### Participants and Site

**Figure 1:**
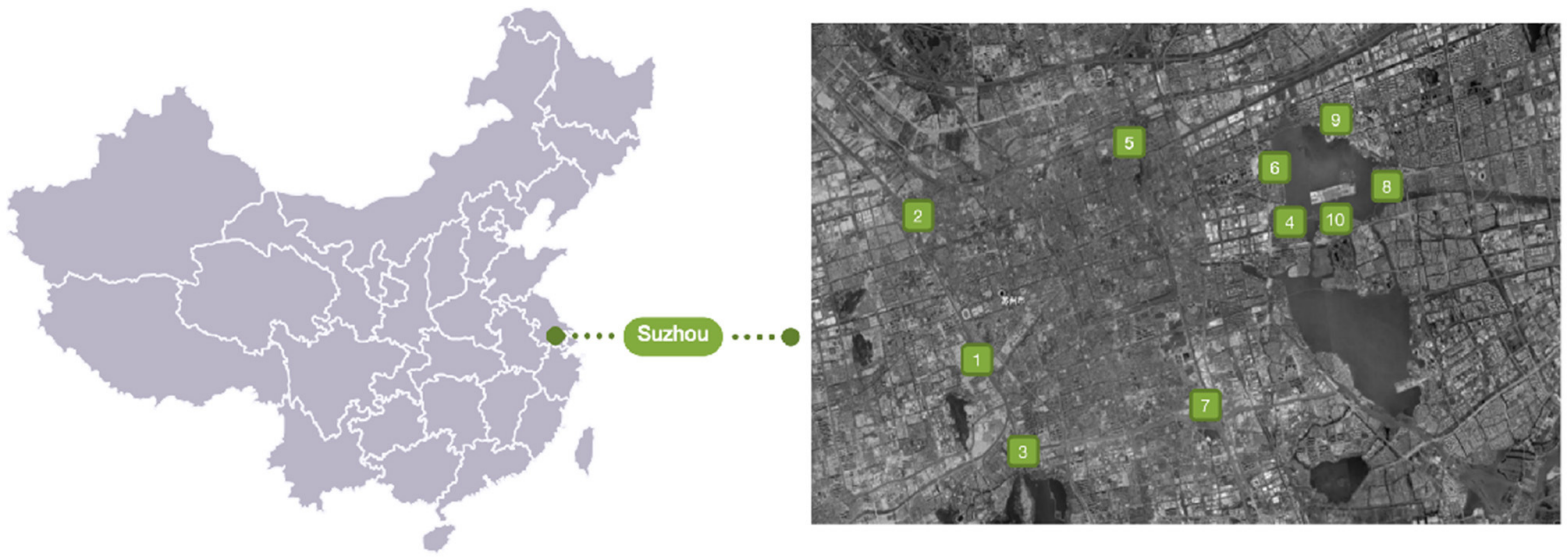
Site locations.

A total of 10 GOSs from Suzhou city are selected, which are located either along the river or lake, indicting the decent environmental qualities and view (Figure 1). The GOSs are represented in the form of parks, afflicted green space, and urban forest. They consist of different areas such as playground, square, shelter seating, jogging trail, or pathway, etc., which are attractive to both adults and children. These GOS could be divided into three categories: (1) community level (1–5 ha), (2) district level (6–10 ha), (3) subcity level (>10 ha). The category criteria come from the official guideline for the GOS planning and design. All the GOSs are located in urban area of the city with high density. Suzhou is a large and typical city in China known for its urban development and planning, with a combination of old towns and new districts. All the studied GOS were distributed around river or lake in the old towns and new districts. The data of these GOSs is drawn from the official documents and input into GOS to make the basic dataset.

Regarding the participants, all of them are recruited on the site after obtaining consent. The recruiting criteria includes: (1) they are attending primary school and are affected by the lockdowns due to pandemic, (2) they are able to wear the devices on the waist, (3) they are planning to play and stay in the GOS, instead of just traveling through, (4) they need to stay in the GOS more than 10 min and <180 min. The investigators conduct the survey in the main entrance of the GOS, and need to screen off unqualified children. After obtaining the verbal consent from their guardians, the qualified children would be invited to join the study. If their guardians were not with them, the investigators would call to ensure their consent and full recognition. All the participants or their guardians would answer a web-based questionnaire for their demographic background and visitation pattern. The qualified participants would be provided with the GPS positioner and accelerometer, and are required to return these devices when they leave the GOSs. For each GOS, we prepare 15–25 sets of devices, depending on how many qualified children there are. The surveys were conducted during the last 2 weeks of April 2020, and the collection period was set up at 9:00–12:00 and 14:30–17:30. Eventually, a total of 179 children participated in the study with valid records.

### Statistical Analysis

Descriptive statistics was used to analyze the children's demographic information, use, and GOS characteristics. To compare the differences of visitation and PA patterns across GOS, one-way ANOVA and chi square tests are utilized. Two regression models are established to explain the associations of the use and GOS characteristics. To examine the effect of GOS on PA pattern of children, multivariate regression is performed with the environmental and surrounding characteristics. Multinomial logistic regression is conducted to explore the relationship of GOS characteristics and visitation pattern of children, which included their visitation frequency, visitation comparison with prior COVID-19 situation, and visitation time. The collinearity diagnostics among the independent and control variables are performed before modeling, and the result showed there was no high correlation among the variables (VIF <10). The significance level was set up at 0.05, and the statistical analysis was performed on the platform SPSS 26 (IBM, Armonk, NY, USA).

## Results

### Visiation Pattern of Children During COVID-19

A total of 179 valid records were obtained (Table 1). The average age of participants was 10.11 years old (min = 6, max = 14), and the majority was ranging from 9 to 12 years old (50.84%), following by group of 10 to 12 years old (41.90%). There were more boys (53.63%) than girls (46.37%) among the participants. More than a half of the children (62.6%) were accompanied by their guardians (parents or other legal adults). Most of them came to visit the GOS on foot (41.90%) and 34.64% of them traveled by public transit, indicating that most of them were living nearby or in the normal economic condition. The majority of participants was living within 1 km (68.72%), which was in accordance with the results above. Regarding the visitation pattern, 48.6% of children preferred to visit GOS on weekdays, while those prone to visit on weekends only accounted for 36.31%. Nearly a half of the participants reported higher frequency of visits (49.16%) than before the pandemic, and only a small amount of them showed less visits (27.37%). The majority of children reported to visit the GOS for 1–3 times per week (68.72%), and only 11.73% of them visit less than a week.

**Table 1:** Description of children and visitation pattern.

**Individual information**	**Items**	**Number**	**Percent**
Age	<6	2	1.12%
	7–9	75	41.90%
	10–12	91	50.84%
	>12	11	6.15%
Gender	Male	96	53.63%
	Female	83	46.37%
Presence of guardians	Yes	112	62.57%
	No	67	37.43%
The way come to the GOS	On foot	75	41.90%
	Public transit	62	34.64%
	Private car	42	23.46%
Distance from home	≤1 km	123	68.72%
	>1 km	56	31.28%
Visitation pattern			
Visitation comparison (with prior Covid-19)	More visitations	88	49.16%
	Almost the same	42	23.46%
	Less visitations	49	27.37%
Visitation time	Weekday	87	48.60%
	Weekend	65	36.31%
	Both	27	15.08%
Visitation frequency	Less than once a week	21	11.73%
	Once to third time a week	123	68.72%
	More than third time a week	35	19.55%

### Phsycial Activity Pattern of Children in GOS

**Figure 2:**
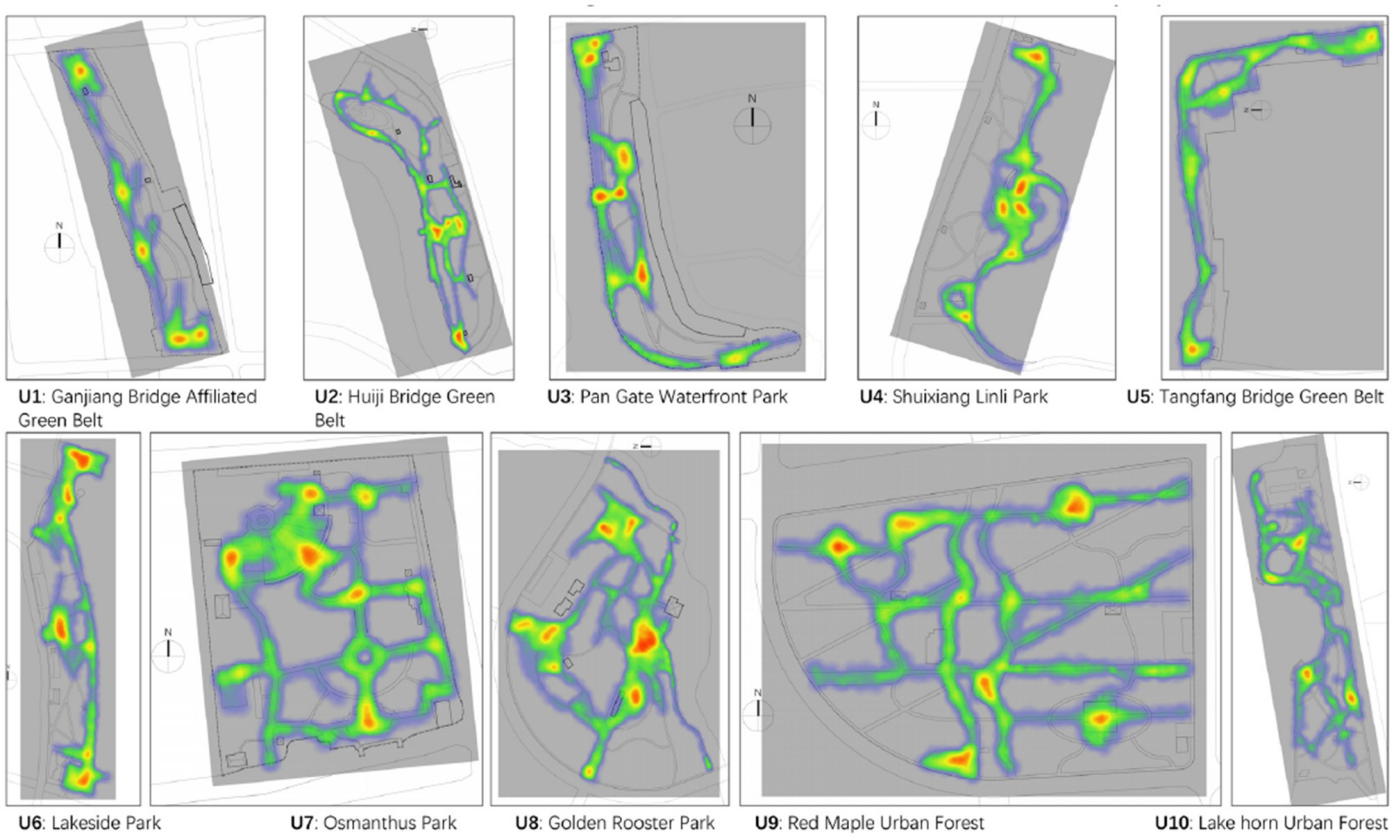
The locations of children's physical activities in the GOS.

Generally, the mean value of walking steps was 2,930.13, with the maximum steps (9,913) and minimum steps (461). The maximum of duration was 129.14 mins, whereas the minimum was 17.13 mins, and the mean value of stay length was 46.70 min. Since we set up a visitation threshold (15–25), the average counts of visiting were 17.90, with a maximum and minimum of 25 and 10, respectively. Regarding the type of GOS, the average walking steps (2,786.52) and duration (46.31 mins) was found in the GOS of subcity level, and then they were followed by district level and community level (Table 2). Regarding the location of PA, the distribution was not even as some places were crowded, and some places showed few records. Generally, children's PA was mainly around a square, playground, sheltered place, and waterside areas (Figure 2). The speed of each records was also categorized into sedentary (<0 m/s), (moderate) 0–1.5 m/s, (vigorous) >1.5 m/s to distinguish different level of PA (Table 3). It was found the proportion of intensive PA go up as the GOS becomes larger.

**Table 2:** The description of visitation and PA pattern by different GOS.

**Children use**	**Community level (1–5 ha)**		**District level (6–10 ha)**		**Sub-City level (>10 ha)**			**Total**
**Visitation pattern**	***n***	**%**	***n***	**%**	***n***	**%**	***p***	**179**
Visitation frequency							0.001^**^	
Less than once a week	6	3.35%	8	4.47%	7	3.91%		21
Once to third time a week	28	15.64%	39	21.79%	56	31.28%		123
More than third time a week	13	7.26%	10	5.59%	12	6.70%		35
Visitation comparison with Prior to COVID-19							0.001^**^	
More visitations	21	11.73%	26	14.53%	41	22.91%		88
Almost the same	10	5.59%	13	7.26%	19	10.61%		42
Less visitations	15	8.38%	16	8.94%	18	10.06%		49
Visitation time							0.082	
Weekday	29	16.20%	36	20.11%	22	12.29%		87
Weekend	9	5.03%	11	6.15%	45	25.14%		65
Both	14	7.82%	7	3.91%	6	3.35%		27
PA pattern	Mean	SD	Mean	SD	Mean	SD		Mean
Duration (mins)	42.12		46.31		51.22		0.001^**^	46.70
Walking steps	2,786.52		2,982.74		3,010.81		0.011^**^	2,901.13

*One-way ANOVA is employed to examine the differences of visitation pattern between different GOS*.

*Chi square test is employed to examine the differences of PA pattern between different GOS*.

^*^p < 0.05,

** *p < 0.01*.

^*^Indicates the significance level.

**Table 3:** Description of GOSs.

**Category**	**Item**	**Participants**				**GPS records**			
			**Speed = 0**		**Speed (0–1.5 m/s)**		**Speed (>1.5 m/s)**		**Total counts**
Community level	U1	10	3,611	61.95%	1,707	29.28%	511	8.77%	5,829
1–5 ha	U2	13	3,715	60.46%	1,863	30.32%	567	9.23%	6,145
	U3	12	3,901	58.04%	2,039	30.34%	781	11.62%	6,721
	U4	22	6,871	64.45%	2,759	25.88%	1,031	9.67%	10,661
District level	U5	15	4,861	63.85%	1,938	25.46%	814	10.69%	7,613
6-10 ha	U6	21	6,788	58.80%	3,247	28.13%	1,509	13.07%	11,544
	U7	23	1,1414	66.24%	3,367	19.54%	2,451	14.22%	17,232
City level	U8	16	4,851	53.95%	2,799	31.13%	1,341	14.91%	8,991
>10 ha	U9	22	6,919	57.89%	3,190	26.69%	1,844	15.43%	11,953
	U10	25	6,797	56.08%	3,415	28.18%	1,908	15.74%	12,120

### Environmental and Surrounding Characteristics of GOS

Regarding the environmental characteristics of GOS, the size ranged from 1.91 to 14.89 ha, with a mean value of 7.64 ha (Table 4). The greenery proportion was all above 60%, which was a baseline for GOS according to official guideline. The average proportion of sports and playground was 13.88%, and it was higher in the subcity level than in the community level. The proportion of facilities and amenities area ranged from 14.23 to 17.87%, with a mean value of 16.27%, which was slightly higher than the proportion of sports and playground. As for the surrounding characteristic, the residential ratio ranged from 30.21 to 42.15%, with a mean value of 36.83%. The intersection density ranged from 43.15 to 22.24/km^2^, with a mean value of 32.64/km^2^. It was evident that the GOS located in the old town showed a higher residential and intersection ratio than that in the new district. The transit density varied with a mean value of 20.20/km^2^, ranging from 25.02 to 12.54/km^2^. Similar with the residential ratio, GOS located in the old town showed a higher transit density than that in the new district. The mixture of land use was calculated by means of entropy index of land uses (49). The mean value was 0.46, and ranged from 0.81 to 0.17, indicating a large variation.

**Table 4:** The GOS environmental and surrounding characteristics.

	**Community GOS**				**District GOS**				**City GOS**		**Total**	
**Environmental characteristics**	**U1**	**U2**	**U3**	**U4**	**U5**	**U6**	**U7**	**U8**	**U9**	**U10**	**Mean**	**(SD)**
Size (ha)	1.91	3.22	3.41	4.71	6.72	7.94	8.92	11.21	13.45	14.89	7.64	4.48
Greenery proportion (%)	61.15	65.14	67.21	69.24	61.34	62.89	67.51	62.31	67.34	66.84	65.10	2.94
Sports and play ground proportion (%)	12.51	14.11	13.64	12.89	13.75	14.12	13.99	14.24	15.34	14.21	13.88	0.78
Facilities and amenities area proportion (%)	17.87	15.32	15.44	14.23	18.15	17.24	15.21	17.54	15.42	16.24	16.27	1.34
Surrounding characteristics												
Residential ratio (%)	42.15	39.21	41.11	38.74	39.52	36.21	35.32	34.56	30.21	31.25	36.83	4.03
Intersection density (n/km^2^)	36.84	42.12	43.15	31.25	39.32	32.15	26.53	22.24	24.61	28.14	32.64	7.42
Transit density (n/km^2^)	20.01	24.52	25.02	25.32	24.21	19.62	19.21	17.21	12.54	14.33	20.20	4.57
Mixture of land use	0.45	0.69	0.77	0.81	0.67	0.32	0.33	0.24	0.17	0.19	0.46	0.25

### The Effect of GOS Characteristics on PA Pattern

The results of one-way ANOVA showed that children in different GOS had significant differences in their average walking steps (*p* < 0.001) and duration (*p* = 0.011). Obviously, the mean value of the two variables was the highest in the city level GOS whereas it was the lowest in the community level (Table 2).

Multivariate regression was performed to further examine the relationship between GOS characteristic and PA pattern of children (Table 5). Walking duration of children was positively associated with the size of GOS (*p* < 0.000), indicating that larger GOS could be more likely attract children to stay longer. Sports and playground proportion was also positively associated with the use duration (*p* = 0.006). It meant that these areas could increase the duration of children's physical activities as well. As for the model of walking steps, it was also positively associated with the size as well as sports and playground proportion.

**Table 5:** Regression of GOS characteristics with PA.

**Dependent variables**	**Walking steps**		**Duration**	
	**B**	***p***	**B**	***p***
Interior characteristics of UGOS				
Size	1.215	0.000^**^	1.233	0.000^**^
Greenery proportion	0.022	0.652	0.042	0.182
Sports and play GROUND proportion	1.475	0.000^**^	0.697	0.006^**^
Facilities and amenities area proportion	−0.028	0.404	−0.022	0.642
Surrounding characteristics of UGOS				
Residential ratio	−0.065	0.091	−0.157	2.631
Intersection density	−0.044	0.182	0.031	0.753
Transit density	0.028	0.718	0.107	0.089
Mixture of land use	−0.037	0.324	−0.427	0.954
Constant	0.877	0.000	0.813	0.000
*R*-square	0.321		0.284	
N	179		179	

^*^p < 0.05,

** *p < 0.01*.

^*^Indicates the significance level.

As for the location of the PA, the geo-data of all the participants were categorized into: (1) Speed = 0, representing that the participants were in the secondary status, (2) Speed > 0, representing that the participants were moving and physically active. In the large GOS, there were more active records, whereas less active records in the small GOS (Table 3). According to the mapping of these records, the locations of PA were not evenly distributed in the GOS (Figure 2). Some places were heavily used whereas some others were seldom occupied. Specifically, places with playing facilities and amenities, landscape, water features, and shaded area were spot with more records. The linear space was long and showed a significantly high percentage of records, such as jogging trail and waterside pathway. Since some children were companied by parents, their locations of PA were usually distributed around squares, water features, and status.

### Associations of GOS Characteristics With Visitation Pattern of Children

The results of Chi-square test showed that participants from different sites showed significant differences in their visitation pattern of frequency (*p* < 0.000) and comparison with prior to COVID-19 (*p* < 0.000). It was clear that the children would like to visit larger GOS more frequently than smaller ones. Similarly, larger GOS could attract more visitation than smaller ones.

Multinomial logistic regression model was used to examine the association of children's visitation pattern and GOS characteristics (Table 6). Results showed that size was significantly associated with visitation frequency (*p* < 0.000) and comparison with prior COVID-19 (*p* < 0.000). The residential ratio was closely associated with visitation comparison with prior COVID-19 (*p* < 0.000) and visitation time (*p* < 0.000). Intersection density was significantly associated with visitation frequency (*p* < 0.000).

**Table 6:** Regression of GOS characteristics with visitation pattern.

**Dependent variables**	**Visitation frequency**		**Visitation comparison with prior COVID-19**		**Visitation time**	
	**B**	***p***	**B**	***p***	**B**	***p***
Environmental characteristics						
Size	9.441	0.000^**^	5.125	0.000^**^	0.205	0.221
Greenery proportion	1.309	0.157	0.816	0.462	0.812	0.057
Sports and play ground proportion	0.779	0.431	0.698	0.783	0.003	0.701
Facilities and amenities area proportion	1.127	0.557	0.902	0.664	0.312	0.375
Surrounding characteristics						
Residential ratio	0.874	0.750	3.481	0.000^**^	7.221	0.000^**^
Intersection density	4.175	0.000^**^	0.789	0.574	−0.126	0.082
Transit density	1.342	0.203	0.561	0.978	−0.496	0.061
Mixture of land use	1.351	0.083	0.747	0.127	0.002	0.407
Constant	−6.411	0.002	−6.111	0.003	−5.876	0.003
Cox and snell R square	0.214		0.257		0.311	

^*^p < 0.05,

** *p < 0.01*.

^*^Indicates the significance level.

## Discussion

This study examines the associations of GOS characteristics with children's use during COVID-19 using site-based survey, GPS, and accelerometer. In particular, it is to explore how the neighborhood and environmental characteristic affect the visitation and PA pattern of children in the GOS in a highly dense urban setting. The results show that the pandemic has posed some changes on children's use, and it could be affected by GOS characteristics. The findings suggest the importance of GOS characteristics from both environmental and surrounding. Necessary environmental internecions of design and planning are needed to promote the children's use for their well-being during and after the pandemic.

### Children Visitations Pattern During COVID-19

The pandemic exerted a great impact on the children's visitation pattern on GOS. Nearly half of the children showed more visitation than before, whereas only a small amount of them showed less visitation. This trend was particularly clear among the groups visiting subcity GOS. This finding might contradict with some previous assumptions that the usage of GOS could be compromised due to the implementation of policies such as social distancing and closure of some public places (8, 10). Actually, some studies asserted the importance of GOS to human health during the pandemic, and observed increasing visitation in certain settings (50). The possible reasons could be that children had more free time due to the lockdown of schools. Many adults had to work at home, which allowed them more time to accompany their children to visit GOS. Moreover, the study was conducted in a highly dense urbanized city, where GOS was a scarce outdoor recreational resource because the majority of urban dwellers lived in a flat or apartment, and they did not have private green space. Another finding was that participants preferred to visit GOS on weekdays than on weekends. It could be different from that before COVID-19, when most of children would go to GOS at the weekend and stay at school at the weekdays. The possible reason could be the lockdown policy which allowed more free time for both children and parents during the weekdays. Besides, the fear of the crowdedness and contact could also discourage children to use GOS during the weekend. In the survey, many of the parents would instruct their children to go on the weekday to avid crowds. Overall, the visitation pattern of children has been profoundly affected by the pandemic, which could be producing negative influence on their physical fitness, especially for boys (51). It is hence necessary to reflect the relationship of GOS characteristic and children visitation pattern during the pandemic.

### The GOS Charatoristics Associated With Children's Visitation Pattern

Regarding the visitation pattern, the study found that both of the neighborhood and environmental Characteristics could affect the frequency, time, and comparison with prior COVID-19 of visitation. For the neighborhood characteristics, residential ratio and intersection density were identified as the main variables associated with visitation comparison, frequency, and time. It indicated that children's visitation pattern was dramatically affected by the surrounding neighborhood environment, which aligned with previous studies. The residential ratio/proportion of neighborhood would affect the importance children perceived with GOS. In this study, all the GOS were located in the highly dense urban areas, indicating that there were could be an abundance of children living around the GOS. During the pandemic, since the high residential ratio means more possibility of crowdedness in the neighborhood, children were prone to seek more visitations to GOS than before, and avoid going on weekends. Intersection density was an indicator of street connectivity, and was reported to negatively associate with visitation frequency (52, 53). Some studies reported that it was positively associated with children's use of open space and parks because increasing connectivity could make these destinations more accessible (46). The possible reason for the differences could be the sense of transportation safety. Since the outbreak of COVID-19, many children and parents would prefer to visit parks near their homes and travel on foot or bikes. High intersection density was always associated with the high road density as well as the amount of street crossings, which could become a barrier to prevent children from traveling. Regarding the environmental characteristics, only size was associated with visitation frequency and comparison. It indicated that children preferred to use larger GOS, which was consistent with previous studies (50). Larger GOS could be safer and more attractive to children during the pandemic because social distancing is more likely to be implemented. This study further proved that, in a highly dense urban environment, large GOS could be more beneficial to promote children's use. Overall, the findings suggested that the GOS located in the high residential density and less road intersections could be of great significance for children's use.

### The GOS Charatoristics Associated With Children's PA Pattern

The study found that only the GOS environmental characteristics were significantly associated with the PA pattern of children. The variable size was both associated with walking steps and duration of children, indicating the positive impact of large GOS on their walking activity. Studies showed that large park was more likely to stimulate children's PA, especially MVPA, by providing more places, trails, and amenities to encourage children to play for a long time as well as more vigorous PA (54). The findings suggested that larger GOS with much ground for sports and play could make children walk more distance and stay there longer. During the pandemic, large GOS was recommended as places for outdoor activity because of its capacity to practice social distancing and avoid crowdedness (50). In another side, the small GOS was found to limit the space for running fields, and features such as slides and swings that were relevant to children's PA (47). Small GOSs were always simpler and of less diversity, which were less exciting and stimulating than larger ones (55). Accordingly, this work found that children in the subcity level GOS reported 224.29 more steps on average than that in the community level. It was clear that walking steps and duration were mainly associated with environmental characteristics of GOS. The findings suggest that it is necessary to fully explore the potential of the large GOS and expand the existing small ones.

Similarly, the variable of sports and playground proportion was positively associated with the walking steps and duration. Adequate places for recreational activity could be an essential asset for the GOS to attract children. The availability of sports grounds such as football fields and basketball courts were closely reported to closely associate with the PA, especially MVPA. During COVID-19, children were encouraged by the authority to do exercises, sports, and recreational activities outdoors, which were believed to protect the body and limit damage by improving their immunity (50). Hence, it was suggested to enlarge the sports and playground by converting hard-covered places and grasslands to recreational places, on the condition of ensuring safety. Designers should create more spaces for individualized activity in place of group and organized sports. The size of field, courts, and trails might be enlarged. New and expanded recreational infrastructures need the reassessment of capacity to ensure the safety when children were playing (50, 55).

### The GOS Layout and Locations of Children's PA

The study found that the layout of GOS was obviously related to the spatial distribution of children's PA. Children often stayed and played around the sports field, playground, squares, and grassland with cover, according to their location records. The findings aligned with previous studies showing that these areas were the most attractive part of GOS (56, 57). The areas with recreational facilities and natural features could always facilitate more outdoor play for children because they provided abundant attractive places to promote enthusiastic and diverse activities.

Notably, the linear elements of the GOS such as pathway, trails, and waterside were also marked with dense records. Walking would be the most common type of PA for children, and the abundance of walkway or trails in the GOS could stimulate children to walk more. Particularly, the walkway of waterside was significantly marked with dense records, underlining the importance of water features for attracting PA. Previous study shows that water features could stimulate the positive emotion of park users, leading to the possibility of increasing PA (58). The findings suggested the advantages of planning the walkway and jogging trails along the waterside, and connecting the different sports and playground to create network for PA. Overall, except for the quantitative characteristics, the layout of GOS could affect the PA as well, and it could be effective to improve PA by providing a combination of quantitive and qualitive interventions.

### Limitations

This study is based on an Asian city with high urban density, and the results could be limited due to its urban context. Another limitation of this article is the crosssectional study design, which could limit its capacity to fully explore the dynamics of the effect of children's use during COVID-19. The scope is limited to only highly urbanized areas, and the finding may not apply to the suburban and rural areas, which are believed to be more attractive to children for outdoor recreation than urban areas for its low density and rich natural features. Another limitation is the interferential approach, which equipped the participants with the GPS and accelerometer, which could produce the possibility of impacting their vigorous PA by reducing their active engagement in the activity. These influences could be mitigated by just measuring their walking steps and locations. Nonetheless, this bias should be addressed by more comprehensive research methods, such as smart wearable devices. The last limitation is that the visitation outcomes are self-reported instead of objective, which could be vulnerable to a social desirability error. Children's responses to survey and PA status might be influenced by the presence of their guardians because of the importance of parent–child relationship in supporting their activity during the pandemic (59). In the future, studies should be designed to control the influence of their parents by distinguishing the group without parents from those with.

## Conclusions

The pandemic has already transformed the relationship of children and GOS. It may force us to reflect our existing GOS practice as well as the design and management measures to ensure its safety and resilience in these special times. Since many children and parents have to move away from crowded public venues, GOS becomes an important outdoor setting for children's well-being by providing a relatively safe and an attractive place for PA. Understanding the effect and characteristics of GOS on children's use could facilitate decision makers and planners to explore the potential of GOS better in the special time. This study examined the associations of GOS characteristics with children's use during the pandemic. The results show that children's visitation pattern of GOS has been deeply impacted by the pandemic. More children prefer to visit large GOS during the weekdays due to the fear of infection. Their visitation pattern is associated with both environmental and surrounding characteristics of GOS, and the most prominent factors are size, residential ratio, and intersection density. Children's PA pattern is mainly affected by the environmental characteristics including size, sports and playground proportion, and layout. The findings suggest that both the environmental and surrounding characteristics of GOS could be considered into the decision-making and managing process of GOS. The large GOS located with high residential density and street connectivity should be given more priority for PA promotion. Moreover, it suggests enlarging the sports and playground area in the large GOS on condition of ensuring the safety. The walkway, trail, and waterside walk could be utilized to connect the grounds to a composite network for stimulating high-level PA. Overall, GOS is an effective solution to respond to the challenge of children's health from the pandemic by promote the use. This findings could provide evidence for the design guidance and recommendations of health and resilient GOS, which could be beneficial for children's well-being during and post COVID-19 era.

## Data Availability Statement

The data could be available upon reasonable requirements through corresponding author due to the agreement with participants.

## Ethics Statement

Ethical review and approval was not required for the study on human participants in accordance with the local legislation and institutional requirements. Written informed consent to participate in this study was provided by the participants' legal guardian/next of kin.

## Author Contributions

MM: conceptualization, funding acquisition, and writing—original draft. MM and WC: data curation. MM, MA, and ST: methodology. ST, MA, and DD: resources. MA: software. MM and MA: writing—review and editing. All authors contributed to the article and approved the submitted version.

## Funding

This research was funded by National Natural Science Foundation of China (Grant No. 5200081561); Fellowship of China Postdoctoral Science Foundation (Grant No. 2021M693729).

## Conflict of Interest

The authors declare that the research was conducted in the absence of any commercial or financial relationships that could be construed as a potential conflict of interest.

## Publisher's Note

All claims expressed in this article are solely those of the authors and do not necessarily represent those of their affiliated organizations, or those of the publisher, the editors and the reviewers. Any product that may be evaluated in this article, or claim that may be made by its manufacturer, is not guaranteed or endorsed by the publisher.
